# Efficacy of acupuncture for psychogenic erectile dysfunction: a randomized, sham-controlled trial

**DOI:** 10.1186/s12610-023-00215-w

**Published:** 2023-12-21

**Authors:** Hao Wang, Xulu Lei, Dongyue Ma, Ziwei Zhao, Anmin Wang, Guanchao Du, Jiwei Zhang, Fu Wang, Jun Guo

**Affiliations:** 1 Department of Andrology, Xiyuan Hospital of China Academy of Chinese Medical Sciences, Beijing, China; 2 Department of Preventive Care Center, Beijing Fengtai Hospital of Integrated Chinese and Western Medicine, Beijing, China

**Keywords:** Acupuncture, Psychogenic, Erectile dysfunction, Sham acupuncture, Sexual dysfunction

## Abstract

**Background:**

Psychogenic erectile dysfunction (pED) is a common sexual dysfunction often accompanied by psychosomatic factors. Its treatment includes oral medications, psychotherapy, and behavioral therapy. Acupuncture’s effect on erectile function in pED patients remains to be investigated. This randomized study evaluated the effects of acupuncture and sham acupuncture in pED patients. Altogether, 66 men with pED were randomized to the acupuncture (n = 33) or sham acupuncture group (n = 33). Both groups have a 6-week treatment with 18 sessions. Primary outcome was the International Index of Erectile Function-5 (IIEF-5) at 6 weeks. Secondary outcomes were IIEF-5 (weeks 2, 4, and 10), erection hardness score (EHS), sexual encounter profile-2 (SEP-2), SEP-3, self-rating anxiety scale (SAS), and self-rating depression scale (SDS).

**Results:**

Among the 66 participants, 64 completed the outcome measurements at week 10. Both acupuncture and sham acupuncture groups had improved IIEF-5 and EHS and decreased SAS and SDS post-treatment (*p* < 0.05). The acupuncture group had significantly better improvement in IIEF-5, EHS, and SEP-3 and significantly reduced SAS and SDS than the sham acupuncture group (*p* < 0.05). The improvement in SEP-2 post-treatment was not significantly different between the two groups (*p* > 0.05). There were no serious adverse events.

**Conclusions:**

The 6-week acupuncture treatment significantly improved the erectile capacity and psychosomatic status of pED patients.

**Trial registration:**

ChiCTR2200064345 (Chinese Clinical Trial Registry) (https://www.chictr.org.cn/showproj.html?proj=174873).

## Résumé

**Contexte:**

La dysfonction érectile psychogène (DEp) est une dysfonction sexuelle courante, souvent accompagnée de facteurs psychosomatiques. Son traitement comprend des médicaments par voie orale, une psychothérapie, et une thérapie comportementale. L’effet de l’acupuncture sur la fonction érectile chez les patients atteints de DEp reste à étudier. Cette étude randomisée a évalué les effets de l’acupuncture et de l’acupuncture simulée chez les patients atteints de DEp. Au total, 66 hommes atteints de DEp ont été répartis par randomisation dans le groupe d’acupuncture (*n* = 33) ou d’acupuncture simulée (*n* = 33). Les deux groupes ont eu un traitement de 6 semaines avec 18 séances. Le critère de jugement principal était l’indice international de la fonction érectile-5 (IIEF-5) à six semaines. Les critères de jugement secondaires étaient l’IIEF-5 (semaines 2, 4 et 10), le score dedureté de l’érection (EHS), le profil de rencontre sexuelle-2 (SEP-2), le SEP-3, l’échelle d’auto-évaluation de l’anxiété (SAS) et l’échelle d’auto-évaluation de la dépression (SDS).

**Résultats:**

Parmi les 66 participants, 64 ont complété les mesures souhaitées à la semaine 10. Les groupes d’acupuncture et d’acupuncture simulée présentaient une amélioration de l’IIEF-5 et de l’EHS et une diminution du SAS et du SDS après le traitement (*p* < 0,05). Le groupe d’acupuncture a eu une amélioration significativement meilleure de l’IIEF-5, de l’EHS et du SEP-3 et une réduction significative de la SAS et de la SDS par rapport au groupe d’acupuncture simulée (*p* < 0,05). L’amélioration post-traitement du SEP-2 n’était pas significativement différente entre les deux groupes (*p* > 0,05). Il n’y a pas eu d’effets indésirables graves.

**Conclusions:**

Le traitement par acupuncture de six semaines a significativement amélioré la capacité érectile et l’état psychosomatique des patients atteints de dysfonction érectile psychogène (DEp).

**Numéro d’enregistrement de l’essai:**

ChiCTR2200064345 (Registre chinois des essais cliniques) (https://www.chictr.org.cn/showproj.html?proj=174873) Date d’inscription : 3 octobre 2022.

**Mots-clés:**

Acupuncture; psychogène; Dysfonction érectile; Acupuncture simulée; Dysfonction sexuelle

## Background

Psychogenic erectile dysfunction (pED) is a type of erectile dysfunction (ED) characterized by the inability to achieve or maintain an erection sufficient for sexual satisfaction due to nonorganic factors, including depression, anxiety, and marital discord [1]. Recently, specific biomarkers are lacking in pED [2]. An epidemiological survey involving 5210 ambulatory men aged > 40 years from 30 provinces and autonomous regions of China showed an ED prevalence of 40.56% [3], and the proportion of pED patients to ED patients also showed a trend toward a higher percentage [4]. pED is usually caused by unidentified psychological factors, which may include anxiety or depression, lack of sexuality education, psychological trauma of a sexual nature, difficulties in interpersonal relationships, and previous unsuccessful sexual intercourse experiences [5, 6]. Cognitive-behavioral and psychosexual therapies are considered treatment of choice for pED, but they usually require high motivation and willingness from patients to cooperate in treatment, partner support and cooperation, and a long treatment period. The use of phosphodiesterase type 5 inhibitors (PDE5I), such as tadalafil and sildenafil, can improve erectile function to a certain extent, but its long-term use can cause a variety of adverse effects, including dyspepsia, increased heart rate, blood pressure changes, nausea and vomiting, dizziness and headache, and abnormal penile erection [7–9].

Acupuncture is used for therapeutic or preventive purposes, in which needles are inserted into acupoints in the skin and deep tissues, and diseases are cured through the conduction effect of meridians and acupoints, which is an important part of traditional Chinese medicine [10–12]. Acupuncture reportedly can prolong ejaculation time and has a positive effect on the treatment of male sexual dysfunction, such as premature ejaculation [13]. Our previous study showed that acupuncture can improve sexual satisfaction and International Index of Erectile Function-5 (IIEF-5) among ED patients. However, due to the small number of included literature, the efficacy of acupuncture for pED is still inconclusive [14]. Additionally, some studies have limitations in terms of placebo use and the setting of efficacy indicators [15]. Currently, acupuncture is increasingly recognized and accepted by pED patients, but there is still an urgent need for clinical evidence to validate the efficacy of acupuncture in pED. Therefore, we designed this randomized, controlled clinical trial to investigate the efficacy and safety of acupuncture in pED.

## Materials and methods

### Study design

The full trial protocol has been published by Wang et al. [16]. Chinese men aged 20–50 years who had IIEF-5 ranging from 8 to 21 points and a regular sexual partner and a stable sex life, with a frequency of ≥ 1 time/week during treatment were eligible for inclusion. Specific details of case inclusion can be found in our published clinical trial protocol [16]. Our study protocol was approved by the Research Ethical Committee of Xiyuan Hospital, China Academy of Chinese Medical Sciences (2022XLA092-3). All procedures were carried out in accordance with the guidelines stipulated in the Helsinki Declaration. In brief, Participants were randomly assigned to receive acupuncture (*n* = 33) or sham acupuncture (*n* = 33) treatment. Both interventions were applied after randomization lasting for 6 weeks with a frequency of 3 times per week with a follow-up duration of 4 weeks. The study data were blinded to the scale evaluators, outcome information collectors, statistical analysts, outcome evaluators, and participants. Moreover, the participants were informed prior to group assignment that they may be randomly assigned to either the acupuncture or sham acupuncture group. At the end of the last treatment session, all participants were assessed blindly and asked, “What kind of treatment do you think was provided to you?” There were only three answers, which were as follows: “acupuncture treatment,” “sham acupuncture treatment,” or “not sure.” We selected GV20 (Baihui), bilateral PC6 (Neiguan), CV4 (Guanyuan), CV3 (Zhongji), bilateral KI12 (Dahe), bilateral KI3 (Taixi), and bilateral LR3 (Taichong) as our treatment acupoints. For the selection of sham acupuncture, we referred to previous studies using non-meridian and non-acupuncture acupoint method. The specific location and depth of the acupoints are shown in our previous protocol [16]. The assessment included IIEF-5, erection hardness score (EHS), sexual encounter profile-2 (SEP-2), sexual encounter profile-3 (SEP-3), self-rating anxiety scale (SAS), and self-rating depression scale (SDS). We used the CONSORTreporting guidelines [17].

### Study outcome measures

The primary outcome was the IIEF-5 score after 6 weeks of treatment. The secondary outcomes included the IIEF-5 scores obtained at weeks 2, 4, and 10; and the EHS class scores [18], SAS [19], SDS [20], positive response rates to SEP-2 (were you able to insert your penis into your partner’s vagina?), and response rates to SEP-3 (did your erection last long enough for you to have a sexual intercourse?) [21], at weeks 2, 4, 6, and 10. Adverse events as the safety indicators were used to assess the safety of the study.

### Statistical analyses

Based on a previous study, acupuncture had an efficacy rate of 69% as treatment for psychogenic ED [22]. We combined our clinical experience and averaged the results of previous clinical studies in sham acupuncture to estimate the response rate, which was at 30% [15, 23]. On this basis, we adopted a statistical validity test using the software power analysis and sample size (PASS) 2021 (NCSS LLC., Kaysville, UT, USA). In the present study, the α was 0.05, β was 0.1, and power was 0.9, and a sample size of 30 was required for each group. We considered that the shedding rate would be 10%. Finally, a total of 66 cases should be included in our study.

Statistical analysis was conducted using statistical product and service solutions (version 25.0, Chicago, IL, USA) software for data analysis. The test level α of 0.05 was used to indicate statistically significant differences at *P* < 0.05. For measurement information, if it conformed to the normal distribution, it was expressed using `x ± s, and the comparison within two groups was performed using the paired samples t-test, and the comparison between two groups was performed using the independent samples t-test. For count data, frequencies (%) were used, and the chi-square test was used in the case of unordered categorical data, and Mann–Whitney U rank sum test was used for ordered categorical data. For repeated measures data, repeated measures analysis of variance (ANOVA) was used, and if the test of sphericity was not met, the results were corrected by one-way ANOVA “Greenhouse–Geisser.”

## Results

**Fig. 1:**
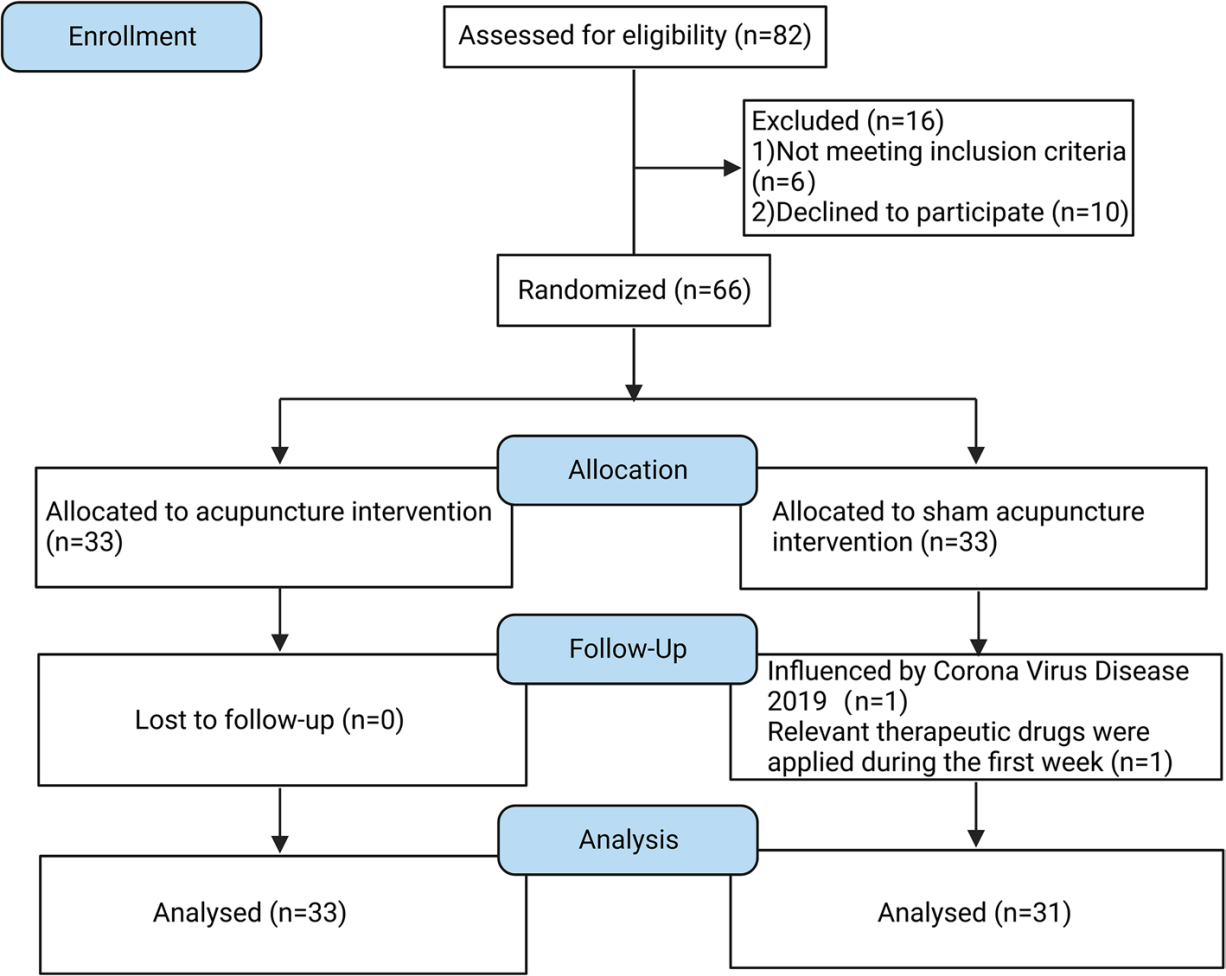
Flowchart of participant recruitment

66 men were included; of these, 64 (acupuncture group = 33, sham acupuncture group = 31) completed the trial. Two participants (sham acupuncture group = 2) were unable to complete the trial; one patient failed to go back to the hospital due to the restrictions during the coronavirus disease pandemic after the first acupuncture treatment; and one patient took medications for ED at 1 week after treatment (Fig. 1). The age, duration of disease, body mass index, educational level, smoking status, alcohol consumption status, marital status, and sexual frequency were not significantly different between the two groups in Table 1 (*p* < 0.05).

**Table 1:** Comparison of the baseline characteristics of patients between the two groups

Characteristic	Acupuncture group (*n* = 33)	Sham acupuncture group (*n* = 31)	*p* value
Mean age (years)	34.76 ± 6.72	35.97 ± 6.98	0.49
Disease duration (months)	14.42 ± 4.11	14.06 ± 3.39	0.96
Body mass index	23.32 ± 1.84	22.95 ± 1.62	0.41
Duration of education (years)	15.91 ± 1.96	15.68 ± 2.36	0.68
Smoker	8(24.24%)	7(22.58%)	0.88
Drinker	5(15.15%)	6(19.35%)	0.66
Married	26(78.79%)	25(80.65%)	0.85
Sexual frequency			0.66
1–2 times per week	27(81.82%)	24(77.42%)	
More than 2 times per week	6(18.18%)	7(22.58%)	

Values are presented as mean ± standard deviation. The statistical analysis are performed via the independent samples t-test and the chi-square test. In the sham acupuncture group, 2 participants were lost to follow up

**Fig. 2:**
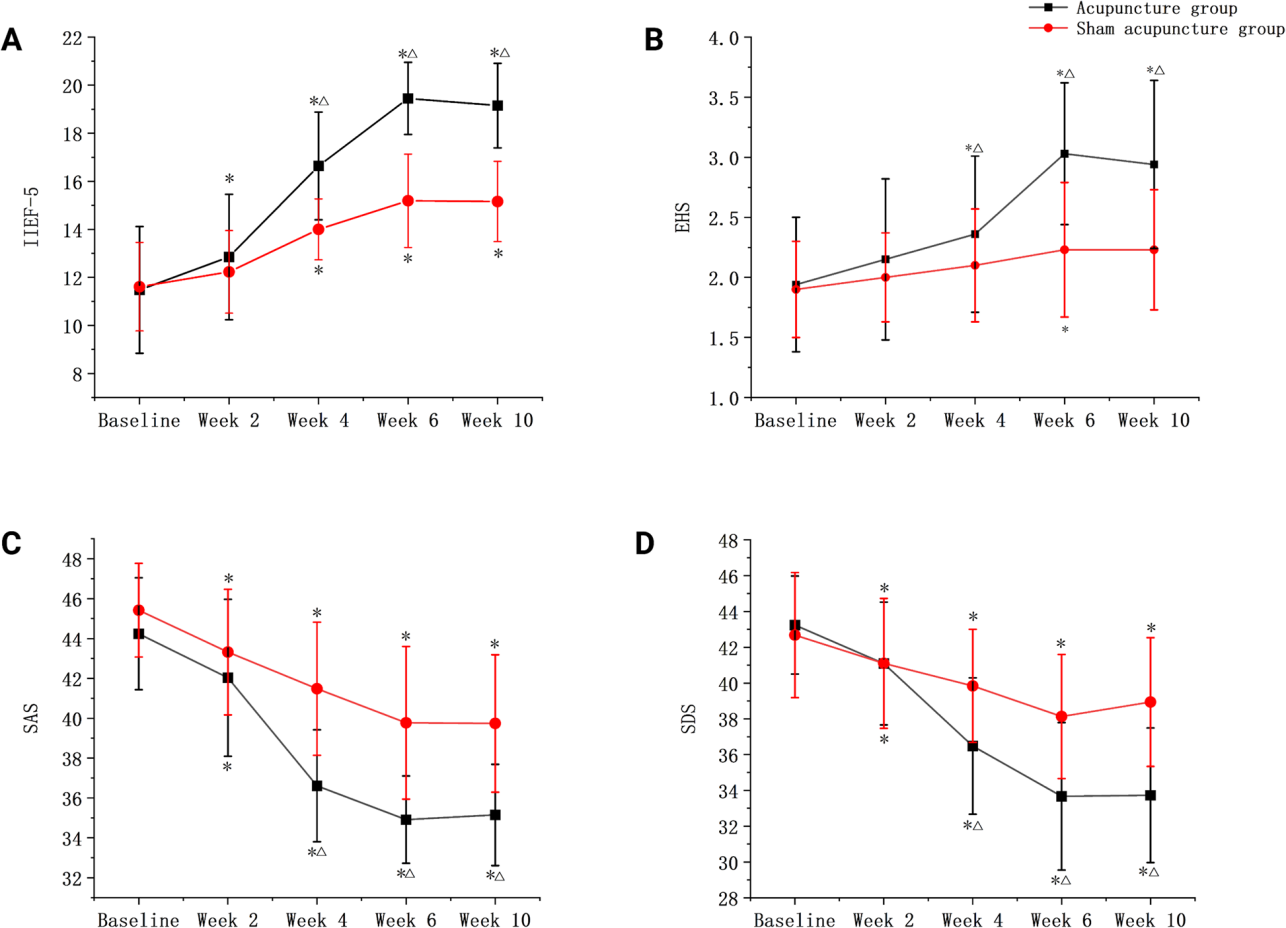
Comparison of IIEF-5, EHS, SAS, and SDS between the acupuncture group and sham acupuncture groups. Abbreviations: IIEF-5, international index of erectile function-5; EHS, erection hardness score; SAS, self-rating anxiety scale; SDS, self-rating depression scale A, Comparison of IIEF-5 between the two groups; B, Comparison of EHS between the two groups; C, Comparison of SAS between the two groups; D, Comparison of SDS between the two groups; * Versus baseline (*p* < 0.05) ^△^Versus sham acupuncture groups (*p* < 0.05). The statistical analysis are performed via the repeated measures analysis of variance

In Table 2 and Fig. 2A, multiple comparisons revealed that the IIEF-5 in the acupuncture group significantly increased sequentially at baseline and at weeks 2, 4, and 6 of treatment (*p* < 0.05); no significant difference was observed in the IIEF-5 between baseline and week 2 of treatment in the sham acupuncture group (*p* < 0.05), but the IIEF-5 from week 2 to weeks 4 and 6 was significantly different (*p* < 0.05). For the primary outcome measure, the difference in the IIEF-5 between week 6 and baseline in the acupuncture group was 7.97 ± 2.66, whereas, in the sham acupuncture group, it was 3.58 ± 2.53, showing significant difference in the t-test of two independent samples (*t* = 6.75, *p* < 0.05), which indicated that at the end of treatment, the efficacy rate of the acupuncture group was significantly better than that of the sham acupuncture group in terms of the improvement of IIEF-5.

**Table 2:** ANOVA results of acupuncture intervention

	Acupuncture group (*n* = 33)	Sham acupuncture group (*n* = 31)	group		time		Group × Time Interaction	
			*F* value	*p* value	*F* value	*p* value	*F* value	*p* value
IIEF-5								
Baseline	11.48 ± 2.68	11.61 ± 1.87	39.30	< 0.001	186.38	< 0.001	25.95	< 0.001
Week 2	12.85 ± 2.65^a^	12.23 ± 1.75						
Week 4	16.64 ± 2.28^ab^	14.00 ± 1.30^a^						
Week 6	19.45 ± 1.52^ab^	15.19 ± 1.97^a^						
Week 10	19.15 ± 1.78^ab^	15.16 ± 1.70^a^						
EHS								
Baseline	1.94 ± 0.56	1.90 ± 0.40	13.99	< 0.001	36.61	< 0.001	11.25	< 0.001
Week 2	2.15 ± 0.67	2.00 ± 0.37						
Week 4	2.36 ± 0.65^ab^	2.10 ± 0.47						
Week 6	3.03 ± 0.59^ab^	2.23 ± 0.56^a^						
Week 10	2.94 ± 0.70^ab^	2.23 ± 0.50						
SAS								
Baseline	44.24 ± 2.85	45.42 ± 2.39	41.14	< 0.001	117.56	< 0.001	11.41	< 0.001
Week 2	42.03 ± 4.00^a^	43.32 ± 3.20						
Week 4	36.60 ± 2.85^ab^	41.48 ± 3.39^a^						
Week 6	34.03 ± 2.57^ab^	39.77 ± 3.90^a^						
Week 10	35.15 ± 2.58^ab^	39.74 ± 3.50^a^						
SDS								
Baseline	43.24 ± 2.78	42.68 ± 3.56	11.93	0.001	103.36	< 0.001	19.00	< 0.001
Week 2	41.09 ± 3.49^a^	41.10 ± 3.69						
Week 4	36.48 ± 3.87^ab^	39.84 ± 3.22^a^						
Week 6	33.67 ± 4.18^ab^	38.13 ± 3.53^a^						
Week 10	33.73 ± 3.82^ab^	38.94 ± 3.66^a^						

*Abbreviations*: *IIEF-5* International index of erectile function-5, *EHS* Erection hardness score, *SAS* Self-rating anxiety scale, *SDS* Self-rating depression scale

Values are presented as mean ± standard deviation. ^a^ Versus baseline ( *p* < 0.05) ^b^ Versus sham acupuncture groups ( *p* < 0.05). The statistical analysis are performed via the repeated measures analysis of variance. In the sham acupuncture group, 2 participants were lost to follow up

In Table 2 and Fig. 2B, the acupuncture group showed a significant increase in EHS at weeks 4, 6, and 10 as compared with the baseline values (*p* < 0.05), and a significant increase in EHS at week 6 as compared with the EHS at week 4 (*p* < 0.05). The sham acupuncture group showed a significant increase in EHS only at week 6 as compared with the baseline values (*p* < 0.05).

Comparing the baseline values of the 2 groups, no statistically significant difference was observed in SEP-2 at weeks 2, 4, 6 and 10 (*p* < 0.05). Regarding the improvement of SEP-3 (Table 3), the differences between the acupuncture group at weeks 4, 6, and 10 compared with the baseline values were statistically significant (*p* < 0.05).

In Table 2, Fig. 2C,D, multiple comparisons revealed that, in the acupuncture group, the SAS and SDS decreased sequentially from baseline to weeks 2, 4, and 6, all reaching significance levels (*P* < 0.05). In the sham acupuncture group, at the beginning of week 2 to weeks 4 and 6, the SAS values decreased sequentially, with the difference being significant (*P* < 0.05). The SDS in the sham acupuncture group decreased significantly at weeks 4, 6, and 10 as compared with the baseline values (*p* < 0.05), whereas no significant difference was observed in the changes between weeks 4, 6, and 10 (*p* < 0.05).

### Blinded assessment

The results of the blinded assessment showed that the implementation of the blinded method was adequate and there was no statistical difference between the two groups in Table 4 (*p* < 0.05).

### Adverse events

During the treatment period, two patients in the acupuncture group experienced post-acupuncture vertigo, which improved after the administration of sugar water. These patients were asked whether they have not eaten breakfast the day of acupuncture, and they were later instructed to only go to the hospital for the acupuncture treatment after eating breakfast. One patient had localized bruising after acupuncture, without bleeding and discomfort; no medical treatment was provided, but the patient was instructed to avoid taking a bath on the same day. The bruising disappeared after 2 weeks. The remaining patients did not experience any obvious adverse reactions during the acupuncture treatments.

## Discussion

**Fig. 3:**
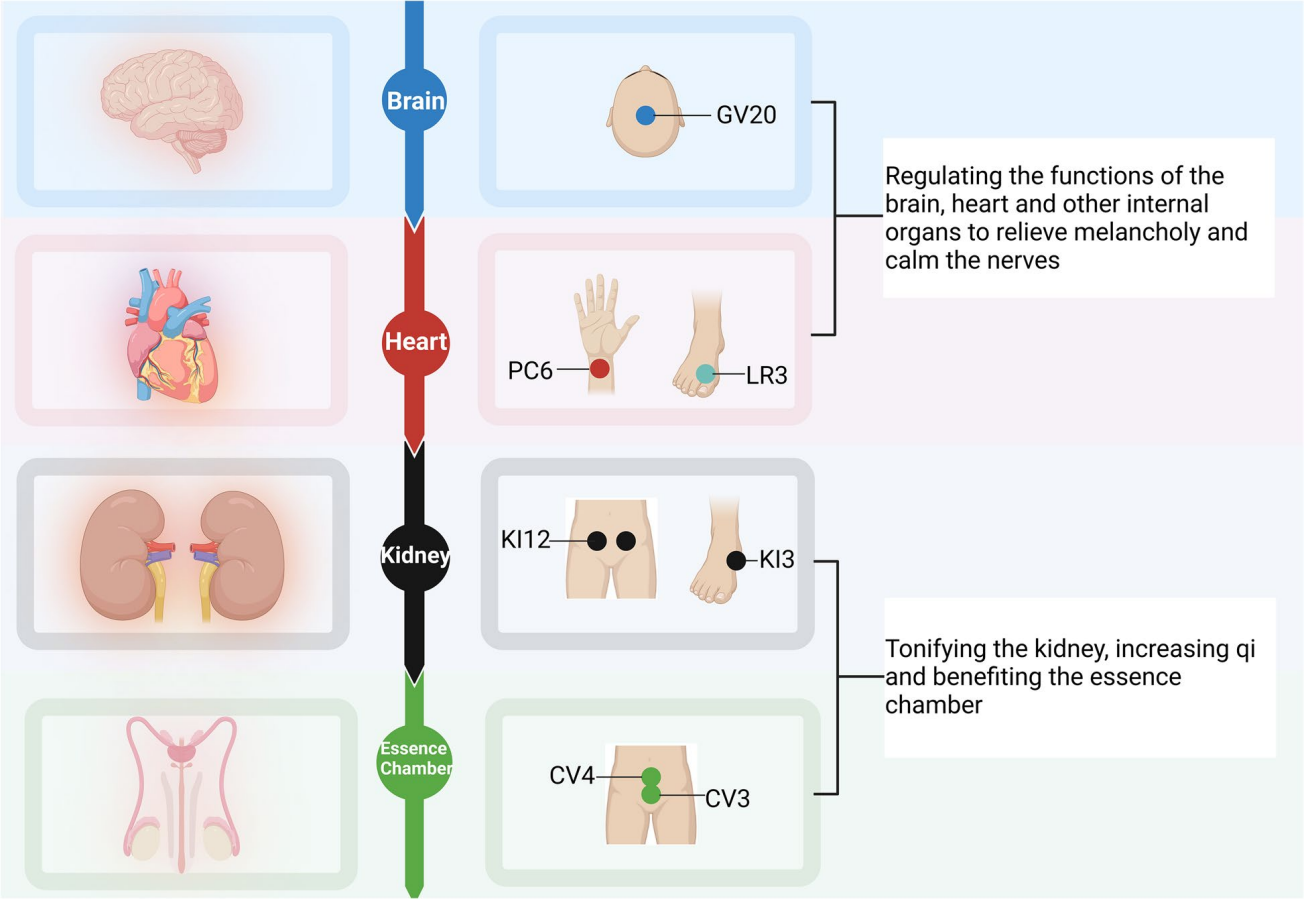
Selection of acupoints for pED based on Chinese medicine theory. Note: From the perspective of traditional Chinese medicine, the essence chamber is a general term for the male reproductive system. GV20 (Baihui) can calm the nerves, PC6 (Neiguan) and LR3 (Taichong) can relieve melancholy, KI12 (Dahe) and KI3 (Taixi) can tonify the kidney, CV3 (Zhongji) and CV4 (Guanyuan) can benefit the essence chamber. Although LR3 (Taichong) is not the acupoint of the heart meridian of hand shaoyin, it is beneficial for regulating mood and mental activity when paired with PC6 (Neiguan). (Created with Biorender.com)

From the perspective of traditional Chinese medicine, pED is related to the dysfunction of brain, heart, kindey, liver and other internal. We previously proposed the application of the "brain–heart-kidney-essence chamber" axis in andrology diseases of traditional Chinese medicine [24], and combined with the theory of traditional Chinese medicine and literature evidence to develop acupoints for the treatment of pED based on this axis [25]. Also, we applied these acupoints in this study (Fig. 3). This randomized controlled trial showed that the 18 sessions of acupuncture treatment for over 6 weeks greatly improved the erectile hardness and quality of sexual life among patients with mild-to-moderate pED as compared to sham acupuncture. The clinical efficacy lasted up to 4 weeks, and acupuncture also improved the patients’ anxiety and depression symptoms as compared with sham acupuncture. Additionally, both acupuncture and sham acupuncture can be performed safely.

In our previous study, we have retrieved four clinical studies on acupuncture as treatment for ED [14]. These studies included two randomized controlled trials and two uncontrolled trials [15, 22, 23, 26]. The results of these studies all indicated that acupuncture or electroacupuncture was effective in the treatment of ED. Engelhardt et al. [15]. evaluated the clinical efficacy of acupuncture in the treatment of 21 pED patients, in terms of the development of a treatment strategy set at 1–2 times/week for 5–20 sessions. The treatment frequency and duration were not specifically standardized, resulting in a possible bias in the trial results of the acupuncture group. Additionally, the acupoints of sham acupuncture group focused on the treatment of headache without setting up blinding. In Aydin et al.'s trial [23], 15 patients with nonorganic ED were treated with electroacupuncture for 6 weeks without mentioning the exact operation. Additionally, the sham acupuncture group chose acupoints that were different from those of the acupuncture group and not relevant to ED treatment. Studies of Kho et al. [26]. and Yaman et al. [22]. did not have a control group. The results of these trials had limitations in terms of the use of sham acupuncture, and these trials might have other limitations because they were conducted without the mention the method of blind and the diagnostic methods for pED; small sample size; lack of specific indicators for efficacy assessment and no additional follow-up.

It is challenging to distinguish the type of ED using IIEF-5 alone [27]. Currently, there is a lack of internationally recognized criteria for pED diagnosis. Clinicians often choose a combination of treatments, including oral PDE5I with audiovisual sexual stimulation (AVSS), intracavernous injection (ICI) combined with color Doppler duplex ultrasonography (CDDU), and nocturnal penile tumescence and rigidity (NPTR) test. NPTR has many limitations, for example, the erections induced by sexual stimulation and those occurring during sleep involve the same blood vessels and structures, but they are regulated by different neural mechanisms [28]. In our study, we performed ICI combined with CDDU to exclud cases of organic ED in patients with abnormal results from oral PDE5I combined with AVSS. Based on careful history taking, we also ruled out related diseases by performing laboratory tests to evaluate for sex and thyroid hormones, among others.

The clinical effect mainly depends on the effective amount of stimulation and sensitivity of the body, whereas the effective amount of stimulation consists of the stimulation intensity and duration; the duration can be controlled from the time cumulative factors, including treatment frequency and duration [29]. In our study, we evaluated the efficacy indicators every 2 weeks during treatment and analyzed them by repeated measures ANOVA. The results showed that over time, the IIEF-5 and EHS scores of the acupuncture group increased sequentially from the beginning of week 2 to week 6, whereas the SAS and SDS scores decreased sequentially from the beginning of week 2 to week 6, and the differences were all statistically significant. The sustained efficacy of acupuncture is that the therapeutic effect still continues to exist for a period of time after the end of acupuncture [30]. Previous studies have confirmed the immediate effect of acupuncture, which plays a major role in the treatment of diseases, especially for acute episodes of diseases [31]. Presently, many male sexual dysfunction diseases are characterized by chronicity, and the continuous effect of acupuncture (post-acupuncture effect) plays an important role in the treatment of andrology diseases [32]. However, none of the previous clinical studies on acupuncture for ED mentioned any follow-up data. To verify the sustained effect of acupuncture in pED treatment, we followed up the patients for 4 weeks, and the results showed that, at 4 weeks after the end of acupuncture treatment, the patients were able to maintain high IIEF-5, EHS, and percentage of “Yes to SEP-3.” Additionally, the SAS and SDS scores can be maintained relatively low as compared to the baseline values. In other words, our study confirmed for the first time the cumulative and lasting effect of acupuncture in pED treatment.

It is interesting to note that the IIEF-5 and EHS at 6 weeks of treatment in the sham acupuncture group were significantly higher than the baseline values, whereas the SAS and SDS scores were significantly lower than the baseline values. Although there was no significant difference in both the SEP-2 and SEP-3 outcomes, this discrepancy suggests a substantial placebo effect of acupuncture on pED. This is consistent with the results of a previous study demonstrating that sham acupuncture itself was associated with effects on sexual dysfunction [13]. Acupuncture is a complex treatment and the placebo effect is inherent in its overall therapeutic efficacy due to the close interaction between the patients, clinicians, and treatment setting [33]. Additionally, unlike previous studies, we used non-meridian and non-acupoint taking in the selection of sham acupuncture, which is a more currently recognized method of sham acupuncture [34]. We also described its specific procedure in our previous protocol [16]. As shown in Table 4, the implementation of the blinded method was adequate, which ensured data reliability. Although sham acupuncture with shallow punctures at non-acupoints has been shown to be effective in assessing the efficacy of acupuncture for certain andrology diseases, it may still have physiologic effects that reduce between-group differences [32].

In our previous literature review, we previously concluded that acupuncture may have a regulatory effect on the hypothalamus, reproductive hormones, local nerves, and the release of nitric oxide in the blood vessels of the penis to treat ED [14]. However, our previous study included patients with different types of ED, and the mechanism of action of acupuncture for psychogenic ED remains unclear. Currently, many studies tend to explore the mechanism of pED from the perspective of brain structure and function [35]. pED patients may have a structural damage to the brain, such as reduced white and gray matter, as well as dysfunctions in multiple regions of the brain [36, 37]. Moreover, most of the abnormalities were on focused on the limbic system and frontal lobes, which have similar neurobiological evidence with depression and anxiety [38]. pED patients may have varying degrees of psychological factors that include anxiety or depressed mood, psychosexual trauma, and pressure resulting from previous unsuccessful sexual intercourse experiences [4, 39]. Although our results showed that the SAS and SDS scores of pED patients at weeks 2, 4, 6, and 10 were significantly decreased as compared to the baseline values, we initially considered that these changes may be associated with restoration of erectile capacity and increased sexual satisfaction. Moreover, we cannot rule out that acupuncture does have a role in regulating anxiety or depression symptoms. Therefore, it is necessary to explore the possible mechanisms of acupuncture in pED from a neurobiological perspective.

### Limitation

The present study has some limitations. First, sham acupuncture might have produced certain physiologic effects. Second, despite our 4-week follow-up, long-term follow-up results are still lacking. Third, due to the small sample size as we have included patients from a single center, we did not conduct a subgroup analysis of patients with mild or moderate pED, stratified according to IIEF-5. Fourth, prior acupuncture exposure might have confounded the results, even though the blinded assessment confirmed its influence to be minimal. Finally, the protocol of performing 18 acupuncture sessions over 6 weeks might be a burden to pED patients in other countries.

## Conclusion

Our findings indicate that acupuncture is more advantageous in improving the quality of sexual life and psychosomatic status of pED patients as compared to sham acupuncture.

## Data Availability

The datasets generated and analysed during the current study are not publicly available due to privacy but are available from the corresponding author on reasonable request.

## Abbreviations

pED: Psychogenic erectile dysfunction
IIEF-5: International index of erectile function-5
EHS: Erection hardness score
SEP-2: Sexual encounter profile-2
SEP-3: Sexual encounter profile-3
SAS: Self-rating anxiety scale
SDS: Self-rating depression scale
AVSS: Audiovisual sexual stimulation
ICI: Intracavernous injection
CDDU: Color doppler duplex ultrasonography
NPTR: Nocturnal penile tumescence and rigidity
